# Association Between Gout and Injury Risk: A National Retrospective Cohort Study

**DOI:** 10.3390/ijerph17103679

**Published:** 2020-05-23

**Authors:** Shih-Hsiang Ou, Chu-Lin Chou, Chia-Wei Lin, Wu-Chien Chien, Te-Chao Fang, Kuo-Cheng Lu, Jin-Shuen Chen

**Affiliations:** 1 Division of Nephrology, Department of Internal Medicine, Kaohsiung Veterans General Hospital, Kaohsiung 813, Taiwan; blueyeou1104@gmail.com; 2 Division of Nephrology, Department of Internal Medicine, Shuang Ho Hospital, New Taipei City 235, Taiwan; chulin.chou@tmu.edu.tw; 3 School of Medicine, College of Medicine, Taipei Medical University, Taipei 110, Taiwan; fangtc@tmu.edu.tw; 4 Department of General Medicine, Shuang Ho Hospital, Taipei Medical University, New Taipei City 235, Taiwan; sky02578@yahoo.com.tw; 5 Department of Medical Research, Tri-Service General Hospital, National Defense Medical Center, Taipei 114, Taiwan; chienwu@ndmctsgh.edu.tw; 6 Division of Nephrology, Department of Internal Medicine, Taipei Medical University Hospital, Taipei 110, Taiwan; 7 Division of Nephrology, Taipei Tzu Chi Hospital, Buddhist Tzu Chi Medical Foundation, Hualien 970, Taiwan; Kuochenglu@gmail.com; 8 School of Medicine, Buddhist Tzu Chi University, Hualien 970, Taiwan; 9 Department of Medical Education and Research, Kaohsiung Veterans General Hospital, Kaohsiung 813, Taiwan; 10 School of Medicine, National Defense Medicine, Taipei 114, Taiwan

**Keywords:** gout, injury, urate-lowering therapy

## Abstract

The association between gout and injury remains unclear. This study aimed to investigate the injury risk in patients with gout. Using the Longitudinal Health Insurance Database (LHID) from 2000 to 2010 in Taiwan, patients with gout (group CFG) and those without gout (group C) were enrolled for further analysis. The CFG group was separated into two subgroups (with and without medication) to determine whether the risk of injury was reduced with drug intervention. The follow-up period was defined as the time from the initial diagnosis of gout to the date of injury. A total of 257,442 individuals were enrolled in this study, with 85,814 people in group CFG and 171,628 people in group C. Using Cox regression analysis, group CFG showed a significant increase in the risk of injury. Traffic injuries, poisoning, falls, crushing/cutting/piercing injury, and suicides were prominent among these injuries. Furthermore, when urate-lowing drugs were used to treat the CFG group, there were no significant differences in the occurrence of injury. Patients with gout had an increased risk of injury overall, and drug intervention did not lower the risk of injury in these patients.

## 1. Introduction

Gout adversely affects joints by the deposition of monosodium urate monohydrate crystals and results in inflammatory arthritis. According to the National Health Research Institutes (NHRI), the prevalence of gout in Taiwan is 4.92%, significantly higher than that in other countries [1]. Patients with gout frequently suffer from multiple comorbidities, including hypertension (HTN), type II diabetes mellitus (DM), cardiovascular disease (CVD), chronic kidney disease (CKD), inflammation, dyslipidemia, and metabolic syndrome [2] in combination with Parkinson’s disease and pre-eclampsia [3].

In most countries worldwide, the occurrence of injury has gradually declined. However, injuries in general, increase morbidity and mortality and result in a huge expenditure for health insurance systems [4]. In Taiwan, the prevalence and incidence of injuries are also decreasing, but they play a major role in contributing to patient death. Injuries can be intentional or unintentional. Risk factors for the occurrence of injuries include chronic diseases (e.g., DM, CKD, orthostatic hypotension, cataracts) and lifestyle factors (e.g., walking stick, sleep duration, mental health) [5]. However, few studies have investigated the relationship between gout and the occurrence of injury.

Approximately one third of patients with incident gout have a comorbidity at diagnosis, such as DM or CKD [6]. Patients who have DM with peripheral neuropathy and neuropathic pain may display greater gait variability, contributing to falls and subsequent injuries [7]. CKD is associated with a decline in muscle mass, strength, and function, and may therefore increase the risk of subsequent injuries [8]. We hypothesized that the onset of gout would increase the risk of injuries, but few studies exist in this area. Therefore, the present study aimed to investigate whether gout directly increases the risk of injury in the Taiwanese population using the LHID from NHRI. Additionally, we examined if drug intervention lowers the risk of injury.

## 2. Materials and Methods

### 2.1. Data Collection

The LHID, established by the NHRI in Taiwan, collected data from subjects enrolled in the National Health Insurance (NHI) program. This was implemented in 1995. The present study collected data randomly selected from a cohort of 1 million subjects covered by the NHI. No statistical significance was observed in the distributions of age and sex between the cohorts in the LHID and the Taiwan NHI enrollees. The disease and diagnostic codes recorded in the registry of clinical visits and hospital care were based on the International Classification of Diseases, Ninth Revision, Clinical Modification (ICD-9-CM). To maintain the privacy of subjects involved in the database, NHRI released data with encoded identification numbers; therefore, personnel without authorization were unable to reveal or link any direct information to the enrollees. This study (IRB number: TSGH IRB no. 2-105-05-082) was approved by the Ethics Review Board of the National Defense Medical Center and supervised in accordance with the tenets of the Declaration of Helsinki (1975) and its later amendment (2013). The requirement of informed consent was waived.

### 2.2. Study Design

To evaluate the risk of injury in patients with gout, an LHID from the year 2005 (LHID 2005) was used. This contained the registration and original claim data of 1,000,000 beneficiaries, randomly sampled from the Registry for Beneficiaries (ID) of the NHIR database from 1 January 2005, to 31 December 2005. Two groups were enrolled, and subjects were divided into those with gout (group CFG) and those without (group C). The inclusion criteria of group CFG were patients aged ≥18 years with a newly confirmed diagnosis of gout (ICD-9-CM 274.XX) between 2000 and 2010 and those visiting outpatient clinics at least thrice a year or hospitalized at least once for a confirmed diagnosis of gout. The year of gout diagnosis served as the index year. Regarding group C, subjects were randomly selected from LHID enrollees without a history of gout and were twice matched with group CFG according to sex, age, and index year. We used 1:2 matching to increase precision and hence power. Exclusion criteria were as follows: confirmed gout before 2000; diagnosis of hyperuricemia before tracking; occurrence of injury before tracking; sex unknown; and age <18 years. The endpoint was the occurrence of injury before subject withdrawal or the end of the follow-up (31 December 2010). Injuries for outcome measurement were defined as ICD-9-CM 800.XX to 999.XX, which include traffic accidents, accidental falls, and intentional and non-intentional injuries. Codes for the individual injury events includes traffic injuries (ICD-9-CM E800-E849), poisoning (ICD-9-CM E850-E869), falls (ICD-9-CM E880-E888), burns and fires (ICD-9-CM E890-E899), drowning (ICD-9-CM E910), suffocation (ICD-9-CM E911-E915), crushing/cutting/piercing (ICD-9-CM E916-E920), excessive heat (ICD-9-CM E900-E900.9), injury caused by animal (ICD-9-CM E906-E906.9), electric current injury (ICD-9-CM E925-E925.9), other unintentional injuries (ICD-9-CM E870-E879, E900-E909, E951-E949), suicide (ICD-9-CM E950-E959), homicide/abuse (ICD-9-CM E960-E969), and intention unknown (E980-E989). Hazard ratios of various injury causes between groups CFG and C were of great interest and were analyzed. We also used the Injury Severity Score (ISS) to assess the trauma severity of injury. ISS ≥16 is usually classified as major trauma and may result in poorer outcomes.

Confounding factors such as sex, age, comorbidities, resident location, and insurance premium were adjusted accordingly. Individuals with comorbidities before the index date were the study subjects. The comorbidities included diabetes mellitus (DM, ICD-9-CM 250), hypertension (ICD-9-CM 401-405), stroke (ICD-9-CM 430-438), chronic renal disease (CKD, ICD-9-CM 585), coronary artery disease (CAD, ICD-9-CM 410-414), liver cirrhosis (ICD-9-CM 570, 571, 572.1, 572.4, 573.1-573.3), chronic obstructive pulmonary disease (COPD, ICD-9-CM 491, 492, and 496), hyperlipidemia (ICD-9-CM 272.0, 272.2, 272.4), and cancer (ICD-9-CM 140-208). This study also evaluated whether urate-lowering drug intervention or compliance affected the risk of injury, including xanthine oxidase inhibitor allopurinol (Anatomical Therapeutic Chemical (ATC) code M04AA01), febuxostat (ATC code M04AA03), uricosuric agents probenecid (ATC code M04AB01), sulfinpyrazone (ATC code M04AB02), and benzbromarone (ATC code M04AB03)] as well as the uricase agent rasburicase (ATC code V03AF07). Medication compliance was calculated as the medication possession ratio (MPR; sum of days of supply of urate-lowering drugs divided by following up duration). We identified the good medication adherence subgroup as MPR > 80%, the intermittent adherence subgroup as MPR between 40% and 80%, and the poor adherence subgroup as MPR < 40%.

### 2.3. Data Analysis

Continuous variables were expressed as mean ± SD. Normally distributed continuous data between patients with and without gout, were compared by unpaired Student’s t-test/one-way ANOVA; nonparametric continuous data were analyzed by the Mann-Whitney U test/Kruskal–Wallis Test. Categorical variables were compared by χ^2^ analysis with Fisher’s exact correction. Cumulative risks of injury were calculated using the Kaplan–Meier method, and the differences in survival were determined by the log-rank test. Univariate and multivariable Cox proportional hazard regression analyses were used to determine the impact of gout on the occurrence of injury and identify other independent predictive variables for these events. *p* < 0.05 was considered statistically significant. The hazard ratios and confidence intervals (95%) from the Cox regression analyses were used as estimates of relative risk.

## 3. Results

### 3.1. Sample Size

**Figure 1:**
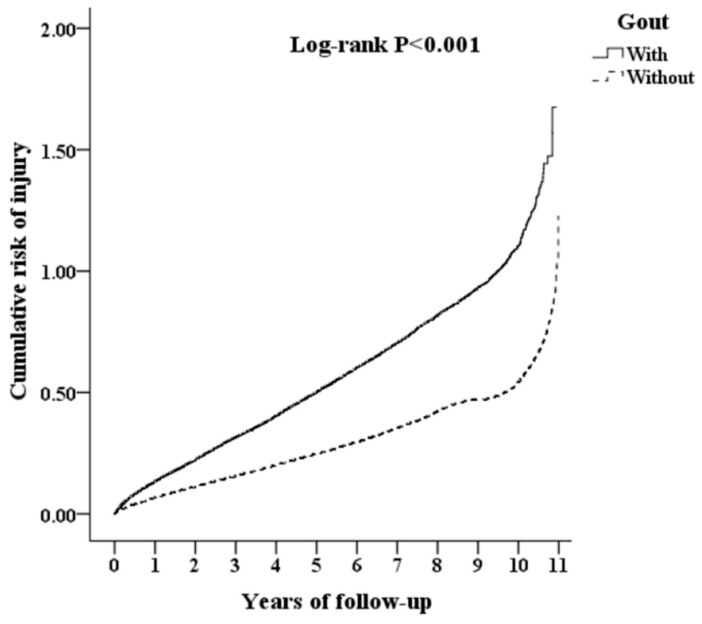
Kaplan–Meier analysis for cumulative risk of injury among patients aged 18 and over stratified by gout with log-rank test.

As showed in Supplementary Figure S1, there was a total of 20,795,043 events from 2000 to 2010 in the Taiwan LHID. Around 986,713 individuals had medical records for visits to outpatient clinics, emergency departments, or hospitalization. Among them, 87,678 were diagnosed with gout, while 1864 were excluded. Overall, the study population totaled 257,442 in this retrospective matched cohort study for analysis, including 85,814 patients with gout (group CFG) and 171,628 without (group C). The incidences of injury in groups CFG and C were 18.92% and 17.83%, respectively, with significant differences (*p* < 0.001) in these two groups, as shown in Figure 1. Therefore, patients with gout had a higher risk of injury.

### 3.2. Baseline Characteristics

Table 1 presents demographics and comorbidities of groups CFG and C at the end of follow-up. The proportion of comorbidities, including DM, HTN, stroke, CKD, and coronary artery disease (CAD), was higher in group CFG than in group C (*p* < 0.001). Additionally, group CFG had a higher proportion of the population living in Central, Southern, and Eastern Taiwan, as well as in the outlying islands than group C (*p* < 0.001). Group CFG had a higher proportion in insurance premiums segment NT $15,841–25,000 than group C (*p* = 0.001).

### 3.3. Differences in Injuries between Groups CFG and C at the End of Follow-Up

Table 2 shows that the risk of injuries in group CFG was significantly higher at the end of follow-up (*p* < 0.001). Further analysis showed that the primary causes of classifiable injuries in both groups were falls, traffic injuries, and other unintentional injuries. In addition, group CFG suffered a more significant proportion of severe trauma when injury occurred (*p* < 0.001).

### 3.4. Risk Factors for the Occurrence of Injuries

As shown in Table 3, after variable adjustment, group CFG had a graver risk of injury (2034 times higher; *p* < 0.001). In addition, subjects with chronic renal disease displayed an enhanced risk of injury (1302 times higher; *p* < 0.001). Furthermore, subjects living in Central, Southern, and Eastern Taiwan, as well as in the outlying islands had a more significant risk of injury than those living in Northern Taiwan (1169–1201 times higher; *p* < 0.001). However, other chronic disease, such as diabetes mellitus, hypertension, stroke, or coronary artery disease, were not a significant risk factor of injury in our study.

### 3.5. Factors for the Occurrence of Injuries by Cox Regression Analysis

A stratified analysis of multiple variables performed to detect the risk of injury in the two groups is shown in Table 4. No statistical difference was found in the male (*p* < 0.001) and female (*p* < 0.001) groups, demonstrating that regardless of sex, the risk of injury was higher in group CFG. Similarly, the risk of injury was higher in group CFG than in group C with or without DM, HTN, stroke, CKD, or CAD (*p* < 0.001).

Additionally, regardless of the season, resident location, or insurance premium, the risk of injury was higher in group CFG (*p* < 0.001). Thus, people with gout had a higher association with the occurrence of injury, independent of other factors.

### 3.6. Relationship between the Occurrence of Subgroup Injuries and Gout

By performing an analysis of subgroups of injury at the end of the follow-up in Table 5, it was found that group CFG had an enhanced risk of traffic injuries, (1575 times higher), poisoning (1650 times higher), falls (1743 times higher), crushing/cutting/piercing (1879 times higher), suicide (1506 times higher), other unintentional injuries (1511 times higher), and not-provided E-code (1473 times higher) compared to group C (*p* < 0.001).

### 3.7. Difference in the Occurrence of Injuries in Group CFG with Medication (M) and without Medication (NM)

**Figure 2:**
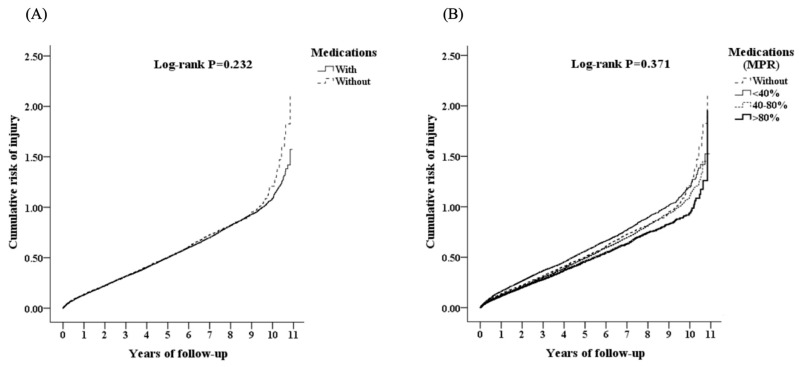
Kaplan–Meier analysis for (**A**) cumulative risk of injury among patients with gout and over, stratified by intervention of medications with the log-rank test and (**B**) cumulative risk of injury among patients with gout and over, stratified by adherence of medications (MPR) with the log-rank test.

Group CFG was separated into two subgroups, those with medication (M) and those without medication (NM), as shown in Supplementary Figure S2. The incidence of injury was 18.76% in subgroup CFG-M and 19.96% in group CFG-NM, without significant differences (*p* = 0.232) between the two groups, as shown in Figure 2A. Group CFG-M was separated into three subgroups using the medication possession ratio (MPR): the incidence of injury in the poor adherence subgroup was 17.00%; in the intermittent adherence subgroup, 20.17%; and in the good adherence subgroup, 19.03%. No significant differences (*p* = 0.371) were found between the three groups in Figure 2B.

## 4. Discussion

To the best of our knowledge, this is the first national population-based cohort study to establish a substantial association between gout and injury. This study confirmed that patients with gout experienced an increased risk of injury. According to our data, four issues were highlighted: first, the enrollment of patients with gout was affected by significant parameters, such as residence, insurance premium, and comorbidity with other chronic diseases; second, risk factors for the occurrence of injuries in Taiwan include gout, chronic renal disease, and resident location; third, in patients with gout, there was a greater incidence of injuries such as traffic injury, falls, and other unintentional injuries; and finally, the intervention of urate-lowering drugs for patients with gout did not lower the risk of the injuries.

In the demographic data, subjects diagnosed with gout in Taiwan displayed significant parameters, such as resident location, insurance premium, and a higher proportion of other chronic diseases. In the Korea multicenter study, summer is the most common season of gout onset [9]. Similarly, a retrospective study in the United Kingdom found that the incidence of gout increased from April to September [10]. In the present study, gout attacks were found to occur particularly in summer and autumn. Regarding the seasonality of gout, previous evidence has proposed seasonal variations of urate, with higher serum levels during the summer months [11]. Changes in diet and increased alcohol consumption, enhanced physical activity and body temperature resulting in dehydration status, may contribute to the development of gout during this period. Regarding location, evidence from Taiwan has suggested that the development of gout maintains regional differences, particularly in the eastern coastal and offshore islands [12]. In the present study, similar findings were presented. A greater incidence of gout was determined particularly in central, southern, and eastern areas and in the outlying islands of Taiwan. As for chronic disease, in the present study, subjects with gout had a significantly higher risk of developing other comorbidities, including DM, HTN, stroke, and CAD. The results were compatible with previous findings from in vivo and in vitro studies, suggesting a strong association with serum uric acid levels, cardiovascular diseases, CKD, and stroke [13,14]. Hyperuricemia may induce oxidative stress [15], stimulate an inflammatory response [16], and cause endothelial dysfunction [17]. A recent study also revealed a connection between hyperuricemia and an increased risk of cardiovascular mortality [18]. In summary, findings in subjects in group CFG display unique correlations with season, residence, and comorbidities; physicians should be alert to these associations when treating patients with hyperuricemia and gout.

In the present study, demographic data and morbidities were analyzed for relationships with the occurrence of injury. The incidence of injury was significantly correlated with patients with gout in terms of chronic renal disease and resident location. Gout is usually associated with long-lasting, abnormally high amounts of uric acid in the blood. It is a painful condition involving the joints, like arthritis. Therefore, a sudden onset of gout may cause gait instability, contributing to falls and subsequent injuries. Another condition, chronic progressive tophaceous arthritis, also causes joint damage, bone destruction and deformity, and may increase the risk of subsequent injuries [19]. Regarding renal disease, injury from falls has been proven to be associated with CKD, particularly in elderly patients undergoing hemodialysis [20]. Polypharmacy, anemia, vitamin D deficiency, sarcopenia, fluid status change, and posture hypotension all potentially contribute to the higher incidence of injury in this group. As we did not further classify the stages of renal diseases in this study, an additional large cohort study is needed to analyze the relationship between CKD stages and the occurrence of injury. Furthermore, regarding resident location, a previous study suggested a significant association among health, lifestyle, and behavior with the development of injury [21]. An inconvenient living environment and poorer medical care is associated with an elevated incidence of injury. Our findings confirmed that location is a risk factor for the occurrence of gout and injuries.

Among the causes of injury, we found that traffic, poisoning, falls, crushing, cutting, piercing, other unintentional injuries, and suicides were significantly higher in people with gout. Patients with gout may display a significant decrease in walking velocity, cadence, and stride length during periods of joint pain. Antalgic gait may increase the risk of traffic injury and falls. When chronic tophi affect the movement and flexibility of the joints, the risk of cutting, piercing, and crushing may also increase. In addition, steroid treatment for gout may induce muscle weakness and osteoporosis and increase the incidence of injury. A recent meta-analysis demonstrated a strong positive relationship between gout and depression [22]. We can reasonably speculate that the greater incidence of suicide and poisoning in gout patients is related to depressive disorders. Our findings emphasize the importance of developing national preventive programs designed to reduce the incidence of injury in patients with gout based on the risk factors.

Intervention with drugs in patients with gout did not lower the risk of injury. Hyperuricemia is the underlying condition accelerating gout and the long-term treatment of it involves the therapeutic lowering of serum and tissue uric acid levels. In 2012, the American College of Rheumatology published its first treatment guidelines. It reasserted that chronic gout needed persistent treatment, and integrated the issues of anti-inflammation, urate-lowering therapy (ULT), and lifestyle risk management [23]. However, in the present study, drug intervention with ULT and drug compliance did not lower the risk of injury in this group. There is a possibility that when subjects were diagnosed with gout, they had already suffered irreversible joint damage because of long-term hyperuricemia-associated comorbidities. After the occurrence of unexpected events, the treatment for gout was inadequate. Further strict randomized control trials are required to explore the real connection and impacts.

There are some limitations to our study. First, the NHIR database does not provide personal history and lifestyle information of patients, such as smoking, drinking, body mass index, and functional capacity. These are the potential confounding factors for this study. In addition, all definitions of variables were based on clinical physicians’ diagnosis and coding; therefore, any misidentification may result in study bias. Second, people with hypertension, diabetes mellitus, stroke, and coronary artery disease did not show an elevated risk of injuries in our studies. This result is unexpected and may differ from previous studies. We believe that this result requires further analysis of the injury subgroup to confirm these findings. Third, we did not analyze the type of medication used in the study or in the comparison cohort due to technical limitations for data retrieval. In addition, we could not evaluate the severity of gout or the interaction with medication use. Fourth, we did not analyze the multiple injury events, which is an important issue in epidemiology studies. Lastly, most injury severity data are unavailable in the NHIR database. Although ISS ≥16 points was employed as the basis to present the severity of patients with trauma, not every injury event was scored.

## 5. Conclusions

Physicians and government policymakers should remain alert to the risk of injury in patients with gout and initiate early therapeutic strategies. The present national population-based cohort study supports the view that gout may directly increase the risk of injury. Although urate-lowing drug intervention did not curtail the incidence of injury in the present study, further research is required to clarify this finding.

## Figures and Tables

**Table 1:** Demographic and Comorbidities of Patients with Gout (Group CFG) and Without (Group C).

	Gout	Total		Group CFG		Group C		*p* Value
Variables		*n*	%	*n*	%	*n*	%	
**Total**		257,442		85,814	33.33	171,628	66.67	
**Sex**								0.999
Male		206,907	80.37	68,969	80.37	137,938	80.37	
Female		50,535	19.63	16,845	19.63	33,690	19.63	
**Age (Years) (Mean ± SD)**		64.15 ± 16.69		63.90 ± 16.13		64.28 ± 16.97		0.041
**Season**								<0.001
Spring (March–May)		62,939	24.45	19,672	22.92	43,267	25.21	
Summer (June–August)		64,590	25.09	21,974	25.61	42,616	24.83	
Autumn (September–November)		68,327	26.54	24,952	29.08	43,375	25.27	
Winter (December–February)		61,586	23.92	19,216	22.39	42,370	24.69	
**Location**								<0.001
Northern Taiwan		101,639	39.48	32,547	37.93	69,092	40.26	
Central Taiwan		73,304	28.47	24,929	29.05	48,375	28.19	
Southern Taiwan		64,734	25.15	21,637	25.21	43,097	25.11	
Eastern Taiwan		16,564	6.43	6208	7.23	10,356	6.03	
Outlying islands		1201	0.47	493	0.57	708	0.41	
**Insured Premium (NT$)**								0.001
<15,840		253,900	98.62	84,517	98.49	169,383	98.69	
15,841–25,000		2832	1.10	1077	1.26	1755	1.02	
≥25,001		710	0.28	220	0.26	490	0.29	
**Diabetes Mellitus (DM)**								<0.001
Without		212,846	82.68	69,797	81.34	143,049	83.35	
With		44,596	17.32	16,017	18.66	28,579	16.65	
**Hypertension**								<0.001
Without		197,472	76.71	56,906	66.31	140,566	81.90	
With		59,970	23.29	28,908	33.69	31,062	18.10	
**Stroke**								<0.001
Without		233,003	90.51	77,098	89.84	155,905	90.84	
With		24,441	9.49	8718	10.16	15,723	9.16	
**Chronic Renal Disease**								<0.001
Without		248,622	96.57	82,486	96.12	166,136	96.80	
With		8820	3.43	3328	3.88	5492	3.20	
**Coronary Artery Disease (CAD)**								<0.001
Without		230,456	89.52	75,336	87.79	155,120	90.38	
With		26,986	10.48	10,478	12.21	16,508	9.62	

*p*-value (category variable: Chi-square/Fisher’s exact test; continue variable: independent t-test); DM: ICD-9-CM 250; Hypertension: ICD-9-CM 401-405; Stroke: ICD-9-CM 430-438; Chronic renal disease: ICD-9-CM 585; CAD: ICD-9-CM 410-414.

**Table 2:** Differences in Injuries between Patients with Gout (Group CFG) and Without (Group C).

	Gout	Total		Group CFG		Group C		*p* Value
Variables		*n*	%	*n*	%	*n*	%	
**Total**		257,442		85,814	33.33	171,628	66.67	
**Injury**								<0.001
Without		210,610	81.81	69,576	81.08	141,034	82.17	
With		46,832	18.19	16,238	18.92	30,594	17.83	
**Cause of injury**								<0.001
Traffic Injuries		6615	14.12	2141	13.19	4474	14.62	
Poisoning		586	1.25	234	1.44	352	1.15	
Falls		10,044	21.45	3322	20.46	6722	21.97	
Burns and Fires		73	0.16	19	0.12	54	0.18	
Drowning		9	0.02	3	0.02	6	0.02	
Suffocation		217	0.46	68	0.42	149	0.49	
Crushing/Cutting/Piercing		1452	3.10	512	3.15	940	3.07	
Excessive Heat		12	0.03	4	0.02	8	0.03	
Injury Caused by Animals		96	0.03	44	0.05	52	0.03	
Electric Current Injury		21	0.04	4	0.02	17	0.06	
Other Unintentional Injuries		9472	20.23	3768	23.20	5704	18.64	
Suicide		555	1.19	179	1.10	376	1.23	
Homicide/Abuse		449	0.96	140	0.86	309	1.01	
Intention Unknown		305	0.65	110	0.68	195	0.64	
No Provided E-Code		16,926	36.14	5690	35.04	11,236	36.73	
**Injury Severity Score (ISS) ≥ 16**								<0.001
Without		257,056	99.85	85,631	99.79	171,425	99.88	
With		386	0.15	183	0.21	203	0.12	

*p*-value (category variable: Chi-square/Fisher’s exact test; continue variable: independent t-test).

**Table 3:** Risk Factors for the Occurrence of Injuries.

Variables	Crude HR	95% CI	95% CI	*p*	Adjusted HR	95% CI	95% CI	*p*
**Gout**								
Without	Reference				Reference			
With	1.992	1.953	2.031	<0.001	2.034	1.995	2.074	<0.001
Sex								
Male	0.912	0.892	0.932	<0.001	0.889	0.869	0.909	<0.001
Female	Reference				Reference			
**Age (Years)**	0.989	0.988	0.990	<0.001	0.993	0.992	0.994	<0.001
**Diabetes Mellitus (DM)**								
Without	Reference				Reference			
With	0.856	0.836	0.876	<0.001	0.894	0.873	0.916	<0.001
**Hypertension**								
Without	Reference				Reference			
With	0.751	0.735	0.768	<0.001	0.787	0.769	0.806	<0.001
**Stroke**								
Without	Reference				Reference			
With	0.618	0.596	0.642	<0.001	0.649	0.626	0.674	<0.001
**Chronic Renal Disease**								
Without	Reference				Reference			
With	1.484	1.422	1.548	<0.001	1.302	1.247	1.360	<0.001
**Coronary Artery Disease (CAD)**								
Without	Reference				Reference			
With	0.612	0.592	0.633	<0.001	0.661	0.639	0.685	<0.001
**Season**								
Spring (March–May)	Reference				Reference			
Summer (June–August)	0.944	0.920	0.968	<0.001	0.932	0.909	0.956	<0.001
Autumn (September–November)	0.813	0.792	0.834	<0.001	0.803	0.783	0.824	<0.001
Winter (December–February)	0.911	0.887	0.935	<0.001	0.919	0.896	0.943	<0.001
**Location**								
Northern Taiwan	Reference				Reference			
Central Taiwan	1.228	1.201	1.256	<0.001	1.169	1.143	1.196	<0.001
Southern Taiwan	1.215	1.186	1.243	<0.001	1.184	1.156	1.212	<0.001
Eastern Taiwan	1.314	1.268	1.361	<0.001	1.201	1.159	1.245	<0.001
Outlying Islands	1.313	1.156	1.492	<0.001	1.174	1.033	1.334	0.014
**Insured premium (NT$)**								
<15,840	Reference				Reference			
15,841–25,000	0.980	0.903	1.063	0.622	0.899	0.828	0.975	0.010
≥25,001	0.718	0.572	0.902	0.004	0.622	0.495	0.782	<0.001

HR = hazard ratio, CI = confidence interval, Adjusted HR: Adjusted variables listed in Table 1. DM: ICD-9-CM 250; Hypertension: ICD-9-CM 401-405; Stroke: ICD-9-CM 430-438; Chronic renal disease: ICD-9-CM 585; CAD: ICD-9-CM 410-414.

**Table 4:** Factors for the Occurrence of Injuries Stratified by Variable Listed in Table 1 by Cox Regression Analysis.

	Gout	Group CFG			Group C			Ratio	Adjusted HR	95%CI	95%CI	*p*
Variables		Event	PYs	Rate (per 105 PYs)	Event	PYs	Rate (per 105 PYs)					
**Total**		16,238	150,342.08	10,800.70	30,594	322,350.39	9490.91	1.138	2.034	1.995	2.074	<0.0011
**Sex**												
Male		12,534	118,736.14	10,556.18	23,960	256,463.93	9342.44	1.130	2.021	1.976	2.066	<0.001
Female		3704	31,605.94	11,719.32	6634	65,886.46	10,068.84	1.164	2.077	1.993	2.165	<0.001
**Diabetes mellitus (DM)**												
Without		13,201	116,597.02	11,321.90	24,862	254,643.69	9763.45	1.160	2.082	2.037	2.127	<0.001
With		3037	33,745.06	8999.84	5732	67,706.70	8465.93	1.063	1.834	1.753	1.919	<0.001
**Hypertension**												
Without		11,736	98,684.39	11,892.46	24,703	238,757.71	10,346.47	1.149	2.000	1.955	2.045	<0.001
With		4502	51,657.69	8715.06	5891	83,592.68	7047.27	1.237	2.184	2.097	2.274	<0.001
**Stroke**												
Without		15,258	134,176.20	11,371.61	28,531	284,544.60	10,026.90	1.134	2.060	2.019	2.103	<0.001
With		980	16,165.88	6062.15	2063	37,805.79	5456.84	1.111	1.667	1.542	1.802	<0.001
**Chronic renal disease**												
Without		15,325	143,313.20	10,693.36	29,281	307,574.55	9519.97	1.123	2.076	2.035	2.119	<0.001
With		913	7028.89	12,989.25	1313	14,775.84	8886.13	1.462	1.492	1.368	1.627	<0.001
**Coronary artery disease (CAD)**												
Without		14,873	129,957.13	11,444.54	28332	284,501.30	9958.48	1.149	2.039	1.998	2.082	<0.001
With		1365	20,384.95	6696.12	2262	37,849.09	5976.37	1.120	1.940	1.810	2.079	<0.001
**Season**												
Spring (March–May)		3967	33,203.35	11,947.59	7760	75,299.86	10,305.46	1.159	2.061	1.981	2.143	<0.001
Summer (June–August)		4271	37,283.12	11,455.59	7792	80,649.66	9661.54	1.186	2.096	2.016	2.178	<0.001
Autumn (September–November)		4326	45,283.44	9553.16	7590	89,206.10	8508.39	1.123	1.985	1.910	2.063	<0.001
Winter (December–February)		3674	34,572.18	10,627.04	7452	77,194.77	9653.50	1.101	1.999	1.919	2.082	<0.001
**Location**												
Northern Taiwan		5326	55,338.61	9624.38	10,761	127,610.68	8432.68	1.141	2.074	2.004	2.145	<0.001
Central Taiwan		5147	44,678.27	11,520.14	9259	91,204.09	10,151.96	1.135	2.082	2.010	2.156	<0.001
Southern Taiwan		4277	37,643.40	11,361.89	8075	79,540.46	10,152.07	1.119	1.967	1.894	2.043	<0.001
Eastern Taiwan		1391	11,782.55	11,805.59	2357	21,562.39	10,931.07	1.080	1.932	1.804	2.069	<0.001
Outlying Islands		97	899.26	10,786.65	142	2432.78	5836.94	1.848	1.779	1.358	2.330	<0.001
**Insured premium (NT$)**												
<15,840		15,975	147,884.48	10,802.35	30,197	317,543.94	9509.55	1.136	2.029	1.989	2.069	<0.001
15,841–25,000		238	2122.27	11,214.41	348	4087.90	8512.93	1.317	2.539	2.136	3.020	<0.001
≥25,001		25	335.33	7455.34	49	718.56	6819.19	1.093	2.946	1.695	5.121	<0.001

PYs = Person-years; Adjusted HR = Adjusted Hazard ratio: Adjusted for variables listed in Cox Regression; CI = confidence interval; DM: ICD-9-CM 250; Hypertension: ICD-9-CM 401-405; Stroke: ICD-9-CM 430-438; Chronic renal disease: ICD-9-CM 585; CAD: ICD-9-CM 410-414.

**Table 5:** Relationship between the Occurrence of Subgroup Injuries in Patients with Gout (Group CFG) and Without Gout (Group C).

	Gout	Group CFG			Group C			Ratio	Adjusted HR	95%CI	95%CI	*p*
Subgroups of Injury		Event	PYs	Rate (per 10 ^5^ PYs)	Event	PYs	Rate (per 10 ^5^ PYs)					
**Total**		16,238	150,342.08	10,800.70	30,594	322,350.39	9,490.91	1.138	2.034	1.995	2.074	<0.001
**Cause of injury**												
Traffic injuries		2141	150,342.08	1424.09	4,74	322,350.39	1387.93	1.026	1.575	1.494	1.861	<0.001
Poisoning		234	150,342.08	155.65	352	322,350.39	109.20	1.425	1.650	1.382	1.970	<0.001
Falls		3322	150,342.08	2209.63	6722	322,350.39	2085.31	1.060	1.743	1.670	1.820	<0.001
Burns and fires		19	150,342.08	12.64	54	322,350.39	16.75	0.754	0.637	0.215	8.189	0.721
Drowning		3	150,342.08	2.00	6	322,350.39	1.86	1.072	1.022	0.674	3.571	0.166
Suffocation		68	150,342.08	45.23	149	322,350.39	46.22	0.979	0.942	0.328	2.554	0.402
Crushing/Cutting/Piercing		512	150,342.08	340.56	940	322,350.39	291.61	1.168	1.879	1.662	2.123	<0.001
Excessive Heat		4	150,342.08	2.66	8	322,350.39	2.48	1.072	1.145	0.976	2.447	0.435
Injury Caused by Animals		44	150,342.08	29.27	52	322,350.39	16.13	1.814	1.976	1.013	3.001	0.026
Electric Current Injury		4	150,342.08	2.66	17	322,350.39	5.27	0.504	0.976	0.432	1.434	0.556
Other Unintentional Injuries		3768	150,342.08	2506.28	5704	322,350.39	1769.50	1.416	1.511	1.126	2.138	<0.001
Suicide		179	150,342.08	119.06	376	322,350.39	116.64	1.021	1.506	1.247	1.820	<0.001
Homicide/Abuse		140	150,342.08	93.12	309	322,350.39	95.86	0.971	0.618	0.301	1.012	0.058
Intention Unknown		110	150,342.08	73.17	195	322,350.39	60.49	1.209	1.447	1.119	1.876	0.005
No Provided E-Code		5690	150,342.08	3784.70	11,236	322,350.39	3485.65	1.086	1.473	1.426	1.552	<0.001
**Injury Severity Score (ISS) ≥16**												
Without		16,055	150,342.08	10,678.98	30,391	322,350.39	9427.94	1.133	2.025	1.986	2.066	<0.001
With		183	150,342.08	121.72	203	322,350.39	62.97	1.933	2.114	1.692	2.641	<0.001

PYs = Person-years; Adjusted HR = Adjusted Hazard ratio: Adjusted for the variables listed in Cox regression in Table 1; CI = confidence interval.

## Supplementary Materials

The following are available online at https://www.mdpi.com/1660-4601/17/10/3679/s1, Figure S1: Flowchart of enrollment of patients with gout (group CGF), matched with those without gout (group C), Figure S2: Flowchart of enrollment of injured patients on medication and those without medication.
