# Supplementary material for: Association Between Gout and Injury Risk: A National Retrospective Cohort Study
Source: Int J Environ Res Public Health. 2020 May 23;17(10):3679. doi: 10.3390/ijerph17103679 (PMC7277708; doi:10.3390/ijerph17103679)
Supplement: Supplementary file 1 [file ijerph-17-03679-s001.zip › Supplement Figure S2.docx]

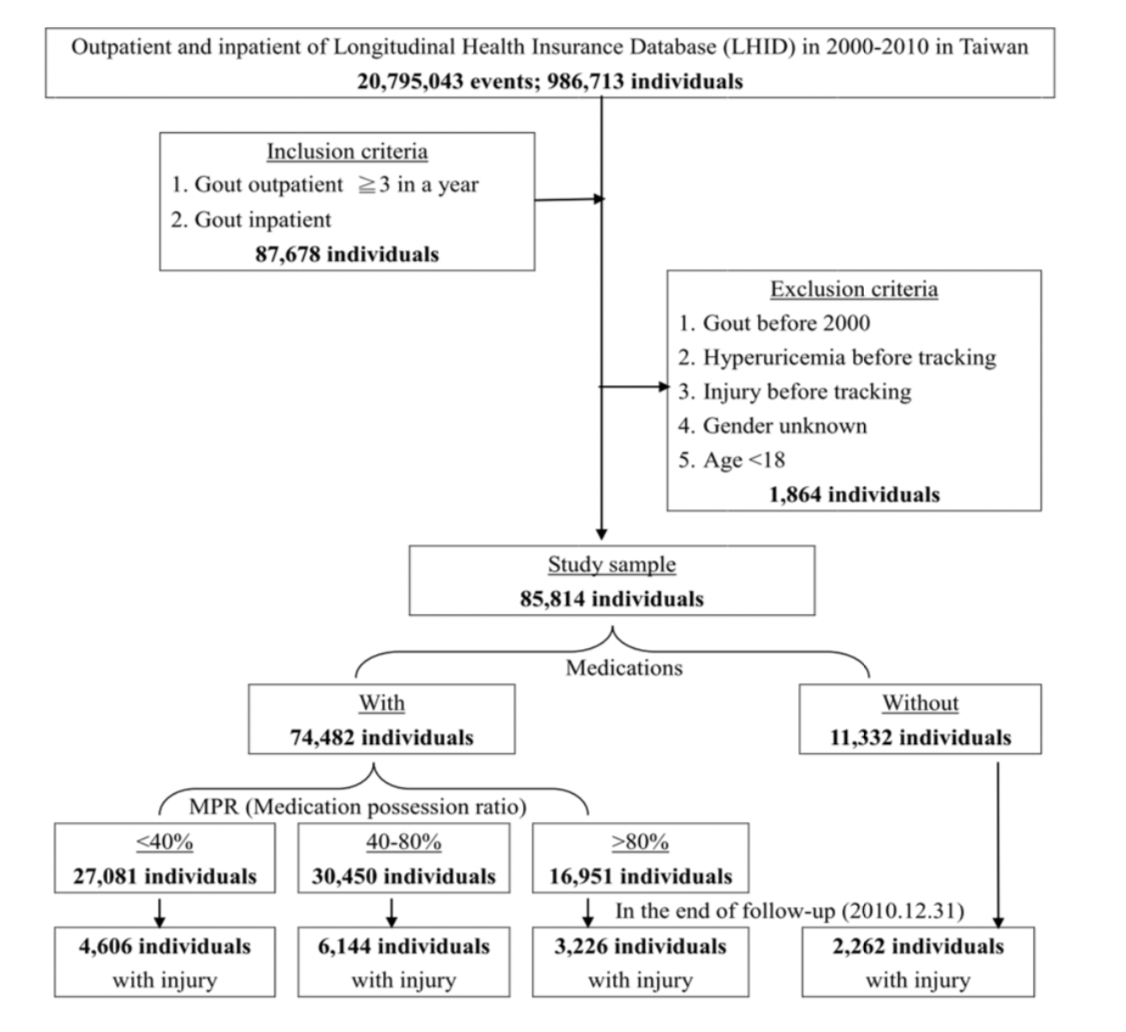


**Supplement Figure S2.** Flowchart of enrollment of injured patients on medication and those without medication.
